# 尼妥珠单抗联合化疗二线及以上治疗晚期肺鳞癌

**DOI:** 10.3779/j.issn.1009-3419.2016.10.05

**Published:** 2016-10-20

**Authors:** 扬 罗, 峻岭 李, 燕 王, 学志 郝, 凤莲 屈

**Affiliations:** 100021 北京，国家癌症中心/中国医学科学院北京协和医学院肿瘤医院内科 Department of Medical Oncology, Cancer Hospital (Institute), National Cancer Center/Cancer Hospital, Chinese Academy of Medical Sciences and Peking Union Medical College, Beijing 100021, China

**Keywords:** 肺肿瘤, 尼妥珠单抗, 联合疗法, Lung neoplasms, Nimotuzumab, Combined therapy

## Abstract

**背景与目的:**

表皮生长因子受体（epidermal growth factor receptor, EGFR）过表达在肺鳞癌中常见，而与预后差相关。本研究旨在观察抗EGFR单克隆抗体尼妥珠单抗联合化疗二线及以上治疗晚期肺鳞癌的疗效及安全性。

**方法:**

回顾性分析13例尼妥珠单抗联合化疗二线及以上治疗的晚期肺鳞癌患者的临床资料，分别采用实体瘤疗效评价标准1.1版和美国国立癌症研究所通用毒性标准4.0版进行疗效和安全性评估。

**结果:**

13例晚期肺鳞癌患者中，1例完全缓解（complete response, CR），2例部分缓解（partial response, PR），4例稳定（stable disease, SD）和6例进展（progressive disease, PD），总有效率（overall response rate, ORR）为23.1%，临床获益率（clinical benefit rate, CBR）为53.8%。6例患者进行了EGFR免疫组化检测，5例EGFR 3+，1例EGFR 2+，这6例患者1例CR，1例PR，4例SD，ORR为33.3%，CBR为100.0%。23.1%的患者出现3度-4度血液学毒性，非血液学毒性轻微，2例（15.4%）出现尼妥珠单抗相关皮疹。

**结论:**

尼妥珠单抗联合化疗二线及以上治疗晚期肺鳞癌有效且不良反应容易耐受，尤其是EGFR表达阳性的患者。

## Abstract (en)

**Background and objective Epidermal:**

growth factor receptor (EGFR) is commonly overexpressed in lung squamous cell carcinoma and has been associated with impaired prognosis. The aim of this study was to observe the efficacy and safety of nimotuzumab, a anti-EGFR monoclonal antibody, combined with chemotherapy as second-or later-line in the treatment of advanced lung squamous cell carcinoma.

**Methods:**

A retrospective analysis of clinical data was conducted in 13 patients with advanced lung squamous cell carcinoma, who were administered with nimotuzumab combined with chemotherapy as second-line or later-line treatment. The efficacy of therapy was evaluated according to Response Evaluation Criteria in Solid Tumors (RECIST) 1.1 and safety by National Cancer Institute Common Toxicity Criteria (NCI-CTC) 4.0.

**Results:**

Of the 13 advanced squamous-cell lung cancer patients, one patient had complete response (CR), 2 patients had partial response (PR), 4 cases had stable disease (SD), and 6 patients had progressive disease. The overall response rate (ORR) was 23.1% and clinical benefit rate (CBR) was 53.8%. EGFR expression were detected by immunohistochemistry in 6 patients and the results showed 5 patients were EGFR 3+ and the other was EGFR 2+. Of these 6 EGFR positive patients, 1 case had CR, 1 case had PR and 4 cases had SD; ORR was 33.3% and CBR was 100.0%. Grade 3/4 hematological toxicities were observed in 3 (23.1%) patients, and non-hametological toxicities were mild. Nimotuzumab-associated skin rash was found in 2 (15.4%) patients.

**Conclusion:**

Nimotuzumab combined with chemotherapy as second-or later-line therapy for advanced squamous cell lung carcinoma was active and well-tolerated, especially for those patients with EGFR positive.

非小细胞肺癌（non-small cell lung cancer, NSCLC）按组织学类型可以分为腺癌、鳞癌和大细胞癌。鳞癌与其他NSCLC组织学类型之间存在肿瘤基因表达谱和免疫学等方面的差异，这些差异导致了现有的一些治疗方案[例如贝伐珠单抗、培美曲塞、表皮生长因子受体酪氨酸酶抑制剂（epidermal growth factor receptor tyrosine kinase inhibitor, EGFR-TKI）]等因为毒性和/或疗效等问题而不适用于鳞癌患者，最新的临床研究表明，接受一线治疗的中晚期鳞癌患者的预后差，与其他NSCLC组织学类型的患者相比，其中位生存期缩短约30%，因此，亟需新的治疗手段。

尼妥珠单抗是一种人源化的抗EGFR的单克隆抗体，有抑制肿瘤细胞增殖和增加化疗敏感性的作用，尼妥珠单抗联合放疗和/或化疗在食管鳞癌，头颈部鳞癌的治疗中取得了良好的效果且耐受性良好^[1, 2]^，因此，我们回顾性分析了2013年1月-2016年4月在中国医学科学院肿瘤医院内科接受尼妥珠单抗联合化疗二线及以上治疗的13例晚期肺鳞癌患者的临床资料，旨在探讨其近期疗效和毒副反应。

## 1 资料和方法

### 1.1 病例选择

2013年1月-2016年4月中国医学科学院肿瘤医院内科收治的使用尼妥珠单抗联合化疗二线及以上治疗的晚期肺鳞癌患者13例。全组患者的中位年龄为59（46-72）岁；男性10例，女性3例。纳入标准：①经组织病理学确诊为肺鳞癌；②经一线化疗后进展的Ⅲb期、Ⅳ期患者；③根据实体肿瘤的疗效评价标准（Response Evaluation Criteria in Solid Tumors, RECIST）1.1，治疗前有可测量的靶病灶；④美国东部肿瘤协作组（Eastern Cooperative Oncology Group, ECOG）全身功能状态评分0分-2分；⑤血常规、肝肾功能基本正常；⑥预计生存时间＞3个月；⑦患者至少完成2个周期的化疗。

### 1.2 治疗方法

13例患者中采用尼妥珠单抗联合铂类为基础的方案10例，尼妥珠单抗联合非铂类单药化疗3例；尼妥珠单抗联合化疗作为二线方案6例，三线方案7例（表 1）。尼妥珠单抗（百泰生物药业有限公司）的用药剂量：1例接受200 mg，其余12例均为400 mg，稀释于250 mL生理盐水中缓慢静脉滴注，给药过程控制在60 min以上，每周给药1次，与化疗同期进行。只有1例患者在尼妥珠单抗联合化疗6周期后获得CR，停止化疗后继续接受尼妥珠单抗联合舒尼替尼维持治疗，肿瘤缓慢进展，肿瘤进展时间22.2个月。3例Ⅲb期患者3个-4个周期尼妥珠单抗联合化疗后接受放疗。

**1:** 13例接受尼妥珠单抗联合化疗治疗的晚期肺鳞癌患者的临床资料 The characteristics of the 13 patients with advanced squamous cell lung cancer who treated with nimotuzumab combined with chemotherapy

No.	Gender	Age (yr)	Stage	Treatment lines	EGFR IHC	Combined CT regimens	Cycles	Response	PFS (mo)
1	Male	48	Ⅳ	2^nd^	NA	PTX+CBP	2	PD	2.4
2	Female	55	Ⅳ	3^rd^	3+	PTX+DDP	6	PR	6.7
3	Male	58	Ⅳ	3^rd^	NA	CPT-11+NDP	2	PD	1.6
4	Female	46	Ⅳ	3^rd^	NA	S1+NDP	4	PD	3.1
5	Female	49	Ⅳ	3^rd^	NA	Nab-PTX	4	PR	2.9
6	Male	62	Ⅳ	2^nd^	2+	PTX+CBP	4	SD	3.9
7	Male	64	Ⅳ	2^nd^	3+	PTX+CBP	4	CR	22.2
8	Male	62	Ⅳ	2^nd^	NA	Nab-PTX	2	PD	1.5
9	Male	64	Ⅲb	3^rd^	3+	PTX+CBP	3	SD	1.6^a^
10	Male	59	Ⅲb	2^nd^	3+	TXT+NDP	4	SD	2.2^a^
11	Male	60	Ⅳ	2^nd^	NA	TXT+DDP	2	PD	1.5
12	Male	55	Ⅲb	2^nd^	3+	TXT+DDP	3	SD	2.5^a^
13	Male	72	Ⅳ	3^rd^	NA	GEM	2	PD	1.8
EGFR: epidermal growth factor receptor; IHC: immunohistochemistry; mo: months; NA: not available; PTX: paclitaxel; CBP: carboplatin; DDP: cisplatin; CPT-11: irinotecan; NDP: nedaplatin; TXT: docetaxel; GEM: gemcitabine; CT: chemotherapy; CR: complete response; PR: partial response; SD: stable disease; PD: progressive disease; ^a^: change to receive radiotherapy.									

### 1.3 疗效评价和不良反应评价

给药后每2个周期进行复查用以疗效评价。按照RECIST 1.1版进行疗效评价，结果分为完全缓解（complete response, CR）、部分缓解（partial response, PR）、稳定（stable disease, SD）和进展（progressive disease, PD）。总有效率（overall response rate, ORR）=（CR+PR）病例/全部病例×100%，临床获益率（clinical benefit rate, CBR）=（CR+PR+SD）/全部病例×100%。根据美国国立癌症研究所通用不良事件术语标准4.0版进行不良反应评价，分为Ⅰ级-Ⅳ级。

## 2 结果

### 2.1 近期疗效评价

末次随访至2016年6月30日，13例患者10例死亡，3例存活。13例患者均可评价疗效，1例CR，2例PR，4例SD，6例PD，ORR为23.1%，CBR为53.8%（表 1）。

7例二线使用尼妥珠单抗联合化疗的患者，1例CR，3例SD，3例PD，ORR为14.3%，CBR为57.1%，6例三线使用尼妥珠单抗联合化疗的患者中，2例PR，1例SD，3例PD，ORR为33.3%，CBR为50.0%。

### 2.2 EGFR免疫组化表达和疗效的关系

13例患者中，6例患者进行了EGFR免疫组化检测，5例EGFR 3+，1例EGFR 2+，这6例患者1例CR，1例PR，4例SD，ORR为33.3%，CBR为100.0%。

### 2.3 不良反应

全组13例患者均可评价不良反应。由尼妥珠单抗引起的皮疹2例，且均为Ⅰ级，发生率为15.4%，无过敏、发热、寒战等：其他不良反应主要为骨髓抑制和胃肠道反应，骨髓抑制主要为白细胞减少，多为可逆性的，无粒细胞减少性发热，无骨髓抑制相关输血，胃肠道反应以恶心、呕吐为主，多为Ⅰ级-Ⅱ级，无毒副作用相关死亡（表 2），这些不良反应多与联合使用的化疗药物相关。

**2:** 尼妥珠单抗联合化疗二线及以上治疗13例晚期肺鳞患者癌的不良反应 Adverse events of 13 patients with advanced squamous cell lung cancer treated to nimotuzumab combined with chemotherapy as second-line or later-line treatment

Symptoms	Incidence rate (%)	Ⅰ	Ⅱ	Ⅲ	Ⅳ
Rash	15.4	2	0	0	0
Leukopenia	92.3	3	6	2	1
Anemia	53.8	6	1	0	0
Thrombocytopenia	23.1	3	0	0	0
Emesis and vomiting	92.3	9	3	0	0
Elevated transaminase	30.8	3	1	0	0

## 3 讨论

20世纪初，肺鳞癌的发病率位居肺癌所有病理类型的首位。从20世纪70年代开始，肺腺癌发病率迅速增加，超过肺鳞癌成为最常见的病理类型。这可能与低焦油过滤嘴香烟使烟草的小分子颗粒被更深吸入到肺部的周边有关，尽管如此，鳞癌仍占NSCLC的25%-30%。

近年来，肺癌相关驱动基因成为研究的热点。有研究报道约60%的肺腺癌患者有驱动基因的突变，其中包括EGFR、棘皮动物微管相关类蛋白4/间变性淋巴瘤激酶（anaplastic lymphoma kinase with echinoderm microtubule-associated protein like 4, *EML4*/*ALK*）融合基因等，相关的靶向治疗药物相继更新换代。而肺鳞癌作为另外一种常见的肺癌类型，却至今没有特异性的分子靶向药物。虽然鳞癌的*EGFR*突变和*ALK*基因重排的发生率小，但是82%的鳞癌存在高*EGFR*基因拷贝数和EGFR蛋白过表达。临床研究^[3-5]^显示EGFR过表达与肿瘤的高侵袭力、高转移性及不良预后高度相关。

尼妥珠单抗属IgGl亚型，可以竞争抑制内源性配体与EGFR结合，阻断EGFR介导的下游信号传导通路，使肿瘤细胞的分裂繁殖受到抑制，同时，还阻断PI3K、AKt、JAK和STAT的信号传导通路，进而阻止肿瘤组织中血管生成，并促使细胞凋亡，增加了化疗敏感性从而起到抑制肿瘤细胞的增殖。尼妥珠单抗具有人源化程度高（高达95%）、亲和力高、选择性高和半衰期长的特点，在治疗中展现出高度的抗肿瘤活性和组织特异性^[6]^。

有临床研究^[7]^显示，尼妥珠单抗单次给药100 mg-800 mg，患者均具备很好的耐受性。Crombet在24例局部晚期头颈部肿瘤中进行了尼妥珠单抗联合放疗的剂量爬坡的Ⅰ期/Ⅱ期临床研究，尼妥珠单抗的剂量分别为50 mg、100 mg、200 mg和400 mg，每周1次，共6周。结果200 mg和400 mg组患者的生存期显著优于低剂量组^[8]^，因此，尼妥珠单抗的常用量为200 mg或400 mg，每周1次，本组患者均采用了200 mg-400 mg的尼妥珠单抗的常规用量。

Babu等^[9]^开展了尼妥珠单抗联合化疗对比单纯化疗治疗晚期NSCLC的多中心开放性随机Ⅱ期临床研究，共纳入110例初治的晚期NSCLC患者，将其随机分入单纯化疗组与尼妥珠单抗联合化疗组，结果尼妥珠单抗联合化疗的ORR显著优于单纯化疗组，ORR分别为54.0%和34.5%（*P*=0.04），且患者耐受性好。亚组分析的结果显示，在肺鳞癌患者中，尼妥珠单抗联合化疗的ORR高达64.3%，提示尼妥珠单抗联合化疗对肺鳞癌的患者疗效更佳。斯晓燕等^[10]^报道了尼妥珠单抗联合化疗治疗晚期肺鳞癌的临床结果，12例患者中，4例PR，6例SD，ORR为25.0%，CBR为83.3%，中位肿瘤进展时间为5.2个月。与本组的结果相比，ORR相仿，CBR和肿瘤进展时间结果优于本组，原因可能为上述研究的患者中包括7例患者为一线治疗，且获得CBR的患者尼妥珠单抗单药维持至进展有关。本组患者均为二线及以上的治疗，绝大多数临床获益的患者未进行尼妥珠单抗的维持治疗，值得注意的是，本组中6例三线治疗的患者中，PR 2例，SD 1例，ORR为33.3%，CBR为50.0%，这提示我们在针对复发耐药的晚期鳞癌的治疗中，可以尝试尼妥珠单抗联合化疗作为抢救性治疗。

尼妥珠单抗作用于EGFR，EGFR的表达能否预测尼妥珠单抗的疗效？Wang等^[11]^做了相应的探索性研究，24例接受尼妥珠单抗联合化疗二线治疗的晚期NSCLC患者中，EGFR荧光原位杂交检测阳性患者的生存期显著延长。另一个关于抗EGFR单克隆抗体西妥昔单抗的FLEX研究入组EGFR表达阳性的初治NSCLC患者，长春瑞滨联合西妥昔单抗组的总生存期优于单纯化疗组，亚组分析显示EGFR高表达的肺鳞癌者的总生存期显著优于肺腺癌及其他病理类型者。这些均提示EGFR和尼妥珠单抗的疗效可能存在一定的相关性。本组中共6例患者进行了EGFR免疫组化检测，5例EGFR 3+，1例患者EGFR 2+，这6例患者的ORR为33.3%，CBR为100.0%，疗效优于不经检测的患者。

西妥昔单抗在国内外的多项临床试验中应用于晚期NSCLC，其Ⅲ级-Ⅳ级的皮疹发生率为11%-22%，且皮疹的发生为剂量依赖性，导致部分患者因为不能耐受重度皮疹而终止用药^[5]^。而尼妥珠单抗的不良反应轻微，不增加化疗的不良反应，尤其皮肤反应发生率低^[12]^，本组共有2例患者出现皮疹，且均为Ⅰ级，与上述文献报道相仿。

尼妥珠单抗联合化疗二线及以上治疗晚期肺鳞癌，不良反应小，患者耐受性好，尤其对EGFR高表达的患者疗效较好。本研究的不足为回顾性分析，且样本量小，但其结果可以对经过多程治疗的晚期肺鳞癌患者提供帮助，并提示有必要进一步扩大样本量用于研究EGFR表达和尼妥珠单抗疗效之间的关系。
